# The effect of three years of TNF alpha blocking therapy on markers of bone turnover and their predictive value for treatment discontinuation in patients with ankylosing spondylitis: a prospective longitudinal observational cohort study

**DOI:** 10.1186/ar3823

**Published:** 2012-04-30

**Authors:** Suzanne Arends, Anneke Spoorenberg, Pieternella M Houtman, Martha K Leijsma, Reinhard Bos, Cees GM Kallenberg, Henk Groen, Elisabeth Brouwer, Eveline van der Veer

**Affiliations:** 1 Rheumatology and Clinical Immunology, University of Groningen, University Medical Center Groningen, P.O. Box 30.001, 9700 RB Groningen, The Netherlands; 2 Rheumatology, Medical Center Leeuwarden, P.O. Box 888, 8901 BR Leeuwarden, The Netherlands; 3 Epidemiology, University of Groningen, University Medical Center Groningen, P.O. Box 30.001, 9700 RB Groningen, The Netherlands; 4 Laboratory Medicine, University of Groningen, University Medical Center Groningen, P.O. Box 30.001, 9700 RB Groningen, The Netherlands

## Abstract

**Introduction:**

The aim of this study was to investigate the effect of three years of tumor necrosis factor-alpha (TNF-α) blocking therapy on bone turnover as well as to analyze the predictive value of early changes in bone turnover markers (BTM) for treatment discontinuation in patients with ankylosing spondylitis (AS).

**Methods:**

This is a prospective cohort study of 111 consecutive AS outpatients who started TNF-α blocking therapy. Clinical assessments and BTM were assessed at baseline, three and six months, as well as at one, two, and three years. Z-scores of BTM were calculated to correct for age and gender. Bone mineral density (BMD) was assessed yearly.

**Results:**

After three years, 72 patients (65%) were still using their first TNF-α blocking agent. In these patients, TNF-α blocking therapy resulted in significantly increased bone-specific alkaline phosphatase, a marker of bone formation; decreased serum collagen-telopeptide (sCTX), a marker of bone resorption; and increased lumbar spine and hip BMD compared to baseline. Baseline to three months decrease in sCTX Z-score (HR: 0.394, 95% CI: 0.263 to 0.591), AS disease activity score (ASDAS; HR: 0.488, 95% CI: 0.317 to 0.752), and physician's global disease activity (HR: 0.739, 95% CI: 0.600 to 0.909) were independent inversely related predictors of time to treatment discontinuation because of inefficacy or intolerance. Early decrease in sCTX Z-score correlated significantly with good long-term response regarding disease activity, physical function and quality of life.

**Conclusions:**

Three years of TNF-α blocking therapy results in a bone turnover balance that favors bone formation, especially mineralization, in combination with continuous improvement of lumbar spine BMD. Early change in sCTX can serve as an objective measure in the evaluation of TNF-α blocking therapy in AS, in addition to the currently used more subjective measures.

## Introduction

Ankylosing spondylitis (AS) is a chronic inflammatory disease that mainly affects the axial skeleton. Bone formation and bone loss are both present in AS. New bone formation can lead to the formation of syndesmophytes, ankylosis of the spine and sacroiliac joints, and bone formations on enthesal sites [1,2], whereas bone loss can result in osteoporosis and vertebral fractures [3-5].

Randomized controlled trials (RCTs) have shown that the tumor necrosis factor-alpha (TNF-α) blocking agents infliximab, etanercept and adalimumab are effective in controlling inflammation and improving clinical assessments in AS [6-8]. Previous studies could not demonstrate a significant effect of two years of TNF-α blocking therapy on radiographic progression in AS [9-11].

Although the majority of patients responds very well, a significant proportion of patients has to withdraw from TNF-α blocking therapy due to inefficacy or adverse events [12-14]. Currently, subjective measures of disease activity, such as the Bath Ankylosing Spondylitis Disease Activity Index (BASDAI) or the global opinion of the physician, are most important in the evaluation of TNF-α blocking therapy in AS. The recently developed Ankylosing Spondylitis Disease Activity Score (ASDAS) captures both subjective (patient-reported measures) and objective (acute phase reactant) aspects of disease activity [14-17]. However, it would be useful to also include a purely objective measure in this evaluation process.

The early effect of TNF-α blocking therapy on bone turnover may be helpful in predicting treatment outcome. Bone turnover can be monitored using bone turnover markers (BTM) [18]. The bone formation markers, bone-specific alkaline phosphatase (BALP) and osteocalcin (OC), were reported to be increased after 2 to 52 weeks and 2 to 22 weeks of TNF-α blocking therapy, respectively [19-21]. On the other hand, the bone resorption markers, serum type I collagen N-telopeptide and C-telopeptide (sNTX and sCTX), remained unchanged up to 46 weeks of TNF-α blocking treatment [19,21,22]. Visvanthan *et al. *showed that an early increase in BALP was associated with significant increases in bone mineral density (BMD) of the spine and hip after two years of TNF-α blocking therapy [23].

The first aim of the present study was to investigate the effect of three years of TNF-α blocking therapy on bone turnover. The second aim was to investigate whether the early effect of TNF-α blocking therapy on BTM can serve as an objective predictor of treatment discontinuation in patients with AS.

## Methods

### Patients

Between November 2004 and December 2007, 111 consecutive Dutch outpatients with AS, who started TNF-α blocking therapy at the University Medical Center Groningen (UMCG; *n *= 28) and the Medical Center Leeuwarden (MCL; *n *= 83), were included in this longitudinal analysis. All patients participated in the Groningen Leeuwarden Ankylosing Spondylitis (GLAS) study, a prospective longitudinal observational cohort study with follow-up visits according to a fixed protocol. For the present analysis, patients with recent fractures and/or use of bisphosphonates were excluded because of major influence on bone metabolism. All patients were over 18 years of age, fulfilled the modified New York criteria for AS (*n *= 109) [24] or the Assessments in Ankylosing Spondylitis (ASAS) criteria for axial spondyloarthritis including MRI (*n *= 2) [25]. The patients started treatment with the TNF-α blocking agents infliximab (*n *= 22), etanercept (*n *= 71), or adalimumab (*n *= 18) because of active disease (BASDAI ≥ 4 and/or expert opinion), according to the ASAS consensus statement [26]. As described previously [14], infliximab (5 mg/kg) was given intravenously at zero, two and six weeks and then every eight weeks. In case of inadequate response, the frequency of infliximab treatment was raised to every six weeks. Etanercept was administered as a subcutaneous injection once (50 mg) or twice (25 mg) a week. Adalimumab (40 mg) was administered as a subcutaneous injection on alternate weeks. In 2004 and 2005, patients started treatment with either infliximab or etanercept as adalimumab was only registered in The Netherlands as of 2006. The choice of the TNF-α blocking agent was based on the judgment of the treating rheumatologist and/or the specific preference of the patient. Continuation of treatment was based on a decrease in the BASDAI of at least 50% or two units compared with baseline and/or expert opinion in favor of treatment continuation. Reasons for discontinuation of TNF-α blocking therapy were classified into the categories: intolerance due to adverse events, inefficacy or other reasons. Patients were allowed to receive concomitant medication as usual in daily clinical practice. The GLAS study was approved by the local ethics committees of the UMCG and the MCL. All patients provided written informed consent according to the Declaration of Helsinki.

### Clinical and laboratory assessments

Patients were evaluated at baseline and after three months (mean 3.3 mo, SD ± 0.5), six months (mean 6.4 mo, SD ± 0.8), one year (mean 1.0 yr, SD ± 0.1), two years (mean 2.1 yr, SD ± 0.1), and three years (mean 3.1 yr, SD ± 0.1) of TNF-α blocking therapy. Disease activity was assessed using the BASDAI (on a scale of 0 to 10), physician's and patient's global assessment of disease activity (GDA; on a scale of 0 to 10), erythrocyte sedimentation rate (ESR), C-reactive protein (CRP), and ASDAS_CRP _(calculated from BASDAI questions 2, 3 and 6, patient's GDA, and CRP) [15,16]. Increased ESR and CRP levels were based on local standardized values. Physical function was assessed using the Bath Ankylosing Spondylitis Functional Index (BASFI; on a scale of 0 to 10). Spinal mobility assessments included chest expansion, modified Schober test, occiput to wall distance and lateral lumbar flexion (left and right). Quality of life was assessed using the Ankylosing Spondylitis Quality of Life questionnaire (ASQoL; on a scale of 0 to 18). Peripheral arthritis was defined as at least one swollen joint (excluding the hip) at baseline.

Bone turnover was studied by assessment of the bone formation markers bone-specific alkaline phosphatase (BALP) and procollagen type 1 N-terminal peptide (PINP), and the bone resorption marker serum type I collagen C-telopeptide (sCTX) [5]. BALP was measured by enzyme-linked immunosorbent assay (ELISA; Metra Biosystems, Mountain View, CA, USA; inter-assay coefficient of variation (IE-CV) 5.5%), PINP by radioimmunoassay (RIA; Orion Diagnostica, Espoo, Finland; IE-CV 9.0%), and sCTX by electrochemiluminescence immunoassay (ECLIA; Elecsys 2010 Roche Mannheim, Germany; IE-CV 10.8%). Serum samples were stored within one hour at -20°C until analysis.

Z-scores of BTM were used to correct for the normal influence that age and gender have on bone turnover. Z-scores, the number of standard deviations (SD) from the normal mean corrected for age and gender, were calculated using matched 10-year-cohorts of a Dutch reference group (200 men or 350 women) checked for serum 25-hydroxyvitamin D levels > 50 nmol/liter as well as for the absence of osteoporosis (BMD T-score > -2.5) after 50 years of age. Z-scores were calculated as follows: (BTM value of individual patient - mean BTM value of matched 10-year-cohort of reference group)/SD of matched reference cohort.

### BMD measurement

BMD of the lumbar spine (anterior-posterior projection at L1 to L4) and hip (total proximal femur) was assessed at baseline and after one year (mean 1.1 yr, SD ± 0.1), two years (mean 2.2 yr, SD ± 0.2), and three years (mean 3.2 yr, SD ± 0.2) of TNF-α blocking therapy. BMD was measured using DXA (Hologic QDR Discovery (UMCG) or Hologic QDR Delphi (MCL), Waltham, MA, USA). T-scores, the number of SD from the normal mean obtained from young healthy adults, and Z-scores, the number of SD from the normal mean corrected for age and gender, were calculated using the NHANES reference database. According to the World Health Organization (WHO) classification, osteopenia was defined as a T-score between -1 and -2.5 and osteoporosis as a T-score ≤ -2.5 [27].

### Statistical analysis

Statistical analysis was performed with PASW Statistics 18 (SPSS, Chicago, IL, USA). Results were expressed as mean ± SD or median (range) for normally distributed and non-normally distributed data, respectively. Predictor analysis of time to discontinuation of TNF-α blocking therapy (yes/no) was performed using forward conditional Cox regression of variables with a *P*-value ≤ 0.3 in univariate Cox regression. The probability of F for entry was 0.05. Receiver Operating Characteristic (ROC) analysis was performed to determine the accuracy of early change in BTM to predict discontinuation of TNF-α blocking therapy during the first three years. Area under the curve (AUC) < 0.70 was interpreted as poor accuracy, 0.70 < AUC < 0.90 as moderate accuracy, and AUC > 0.90 as high accuracy [28]. Pearson's and Spearman's correlation coefficients were used as appropriate to analyze the relation between early change in BTM and clinical assessments. Generalized estimating equations (GEE) were used to analyze clinical assessments, BTM, and BMD over time within subjects. Pairwise contrasts were used to compare baseline and follow-up visits. *P*-values < 0.05 were considered statistically significant.

## Results

The mean age of the 111 AS patients was 42.2 years (SD ± 10.3), 70% were male, and median disease duration was 16 years (range 1 to 49). All baseline characteristics are shown in Table 1.

**Table 1:** Baseline characteristics of the AS study population

Number of patients	111		
Age (yrs)	42.2 ± 10.3		
Gender (male) (n, %)	78 (70)		
Duration of symptoms (yrs)	16 (1 to 49)		
Time since diagnosis (yrs)	9 (0 to 37)		
HLA-B27+ (n, %)	88 (81)		
History of IBD (n, %)	11 (10)		
History of uveitis (n, %)	31 (28)		
History of psoriasis (n, %)	9 (8)		
Peripheral arthritis (n, %)	21 (19)		
Current NSAID use (n, %)	98 (88)		
Current DMARD use (n, %)	26 (23)		
BASDAI (range 0 to 10)	6.1 ± 1.7	BASDAI ≥ 4 (n, %)	98 (88)
ASDAS_CRP_	3.8 ± 0.8	ASDAS_CRP _≥ 2.1 (n, %)	105 (96)
Physician's GDA (range 0 to 10)	5 (1 to 9)		
Patient's GDA (range 0 to 10)	7 (1 to 10)		
ESR (mm/h)	22 (2 to 90)	Increased ESR (n, %)	86 (78)
CRP (mg/l)	15 (2 to 99)	Increased CRP (n, %)	84 (76)
BASFI (range 0 to 10)	6.1 ± 2.0		
Chest expansion (cm)	3.0 (0.5 to 10.0)		
Modified Schober test (cm)	2.9 (0.1 to 7.0)		
Occiput to wall distance (cm)	5.0 (0.0 to 28.0)		
Lateral lumbar flexion L (cm)	8.7 ± 4.1		
Lateral lumbar flexion R (cm)	8.4 ± 4.3		
ASQoL (range 0 to 18)	10 ± 4		
BALP (U/L)	17.2 (1.6 to 36.5)		
BALP Z-score	0.28 (-2.59 to 5.16)		
PINP (μg/l)	45.6 (16.0 to 98.0)		
PINP Z-score	0.35 (-1.75 to 3.63)		
sCTX (pg/ml)	206.0 (13.4-657.1)		
sCTX Z-score	-0.34 (-2.58 to 4.01)		
LS BMD T-score	-0.58 ± 1.41	Osteopenia LS (n, %)	34 (34)
		Osteoporosis LS (n, %)	9 (9)
LS BMD Z-score		LS Z-score ≤ -2.0 (n, %)	9 (9)
Hip BMD T-score	-0.53 ± 1.12	Osteopenia hip (n, %)	38 (37)
		Osteoporosis hip (n, %)	2 (2)
Hip BMD Z-score		Hip Z-score ≤ -2.0 (n, %)	4 (4)

Values are mean ± SD or median (range) unless otherwise indicated.

AS, Ankylosing Spondylitis; ASDAS, Ankylosing Spondylitis Disease Activity Score; BALP, bone-specific-alkaline phosphatase; BASDAI, Bath Ankylosing Spondylitis Disease Activity Index; BASFI, Bath Ankylosing Spondylitis Functional Index; BMD, bone mineral density; CRP, C-reactive protein; DMARD, disease-modifying antirheumatic drug; ESR, erythrocyte sedimentation rate; GDA, global disease activity; HLA-B27+, human leukocyte antigen B27 positive; IBD, inflammatory bowel disease; L, left; LS, lumbar spine;. NSAID, non-steroidal anti-inflammatory drug; PINP, procollagen type 1 N-terminal peptide; R, right; sCTX, serum C-telopeptide of type I collagen

After three years, 72 patients (65%) continued to use their first TNF-α blocking agent. In these patients, all assessments of disease activity (Table 2), physical function, spinal mobility and quality of life (data not shown) improved after three months and remained significantly better compared to baseline up to three years of TNF-α blocking therapy.

**Table 2:** Clinical assessments and bone turnover in AS patients still using their first TNF-**α **blocking agent

Assessment	Baseline	3 months	P*	6 months	P*	12 months	P*	24 months	P*	36 months	P*
BASDAI (range 0 to 10)	6.0 ± 1.7	2.5 ± 1.6	0.000	2.3 ± 1.7	0.000	2.7 ± 2.1	0.000	2.5 ± 1.8	0.000	2.6 ± 1.7	0.000
ASDAS_CRP_	3.9 ± 0.8	1.7 ± 0.7	0.000	1.6 ± 0.7	0.000	1.9 ± 0.9	0.000	1.9 ± 0.8	0.000	1.8 ± 0.8	0.000
Physician's GDA (range 0 to 10)	5 (1 to 9)	2 (0 to 5)	0.000	1 (0 to 8)	0.000	1 (0 to 7)	0.000	0 (0 to 6)	0.000	0 (0 to 3)	0.000
Patient's GDA (range 0 to 10)	6 (1 to 10)	2 (0 to 8)	0.000	2 (0 to 8)	0.000	2 (0 to 10)	0.000	2 (0 to 8)	0.000	2 (0 to 7)	0.000
ESR (mm/h)	22 (2 to 90)	5 (2 to 35)	0.000	6 (2 to 36)	0.000	7 (2 to 52)	0.000	9 (2 to 46)	0.000	8 (2 to 48)	0.000
CRP (mg/l)	16 (2 to 99)	3 (2 to 20)	0.000	3 (2 to 21)	0.000	3 (2 to 38)	0.000	3 (2 to 61)	0.000	3 (2 to 57)	0.000
BALP Z-score	0.19 (-1.64 to 3.91)	0.94 (-1.49 to 5.12)	0.000	0.48 (-1.26 to 4.37)	0.000	0.87 (-1.34 to 6.69)	0.000	0.58 (-1.46 to 6.11)	0.000	1.09 (-1.33 to 9.27)	0.000
PINP Z-score	0.36 (-1.75 to 3.63)	0.50 (-1.54 to 4.55)	0.047	0.54 (-1.47 to 5.33)	0.054	0.28 (-1.52 to 5.53)	0.102	0.57 (-1.51 to 7.17)	0.013	0.28 (-1.45 to 2.43)	0.247
sCTX Z-score	-0.02 (-2.58 to 4.01)	-0.74 (-2.53 to 2.29)	0.000	-0.55 (-2.25 to 3.99)	0.002	-0.45 (-2.23 to 3.11)	0.011	-0.34 (-2.15 to 4.31)	0.119	-0.70 (-2.50 to 4.15)	0.019
LS BMD Z-score	-0.36 ± 1.56					0.04 ± 1.42	0.000	0.20 ± 1.39	0.000	0.48 ± 1.61	0.000
Hip BMD Z-score	-0.40 ± 1.06					-0.32 ± 0.91	0.000	-0.24 ± 1.01	0.000	-0.16 ± 1.03	0.000

Data of the 72 AS patients who were still using their first TNF-α blocking agent after three years were analyzed.

Values are mean ± SD or median (range).

See Table 1 for abbreviations.

* *P*-value compared to values recorded at baseline.

The remaining 39 patients (35%) discontinued treatment after a median follow-up of 7.0 months (range 1.1 to 36.2). Reasons for discontinuation of TNF-α blocking therapy were inefficacy (*n *= 17; 44%), adverse events (*n *= 11; 28%: diarrhea or inflammatory bowel disease (IBD; *n *= 4); infection (*n *= 3); allergic reaction (*n *= 2); cardio-vascular disease (*n *= 1); demyelization problems (*n *= 1)), both inefficacy and adverse events (*n *= 5; 13%: diarrhea or IBD (*n *= 2); infection (*n *= 1); allergic reaction (*n *= 1); uveitis (*n *= 1)), or other reasons (*n *= 6; 15%: good initial response, own choice (*n *= 3); pregnancy wish (*n *= 2); lost to follow-up (*n *= 1)).

### Effect of TNF-α blocking therapy on bone turnover

**Figure 1:**
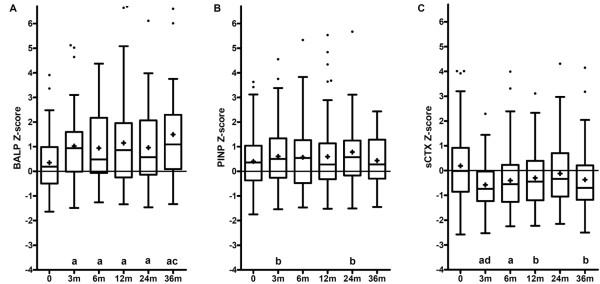
**The effect of three years of TNF-**α **blocking therapy on BTM in patients with AS**. **(A) **bone formation marker BALP. (**B) **bone formation marker PINP. **(C) **bone resorption marker sCTX. Z-scores were calculated to correct for the normal influence that age and gender have on bone turnover. Data were available for 100%, 92.6%, 95.4%, 97.2%, 97.2% and 90.3% of the 72 patients at 0, 3, 6, 12, 24 and 36 months, respectively. Box-and-whisker plots (Tukey): boxes indicate medians with interquartile ranges; + indicate means; whiskers indicate 1.5 times the interquartile distances; • indicate outliers. a: *P *< 0.001 compared to values recorded at baseline. b: *P *< 0.05 compared to values recorded at baseline. c: *P *< 0.05 and *P *< 0.01 compared to values recorded at 6 and 24 months, respectively. d: *P *< 0.05 and *P *< 0.01 compared to values recorded at 12 and 24 months, respectively.

Data of the 72 AS patients who were still using their first TNF-α blocking agent after three years were analyzed to investigate the effect of TNF-α blocking therapy on bone turnover (Table 2). TNF-α blocking therapy resulted in a significant increase in the bone formation marker BALP Z-score after three months (*P *< 0.001) and BALP Z-score continued at a higher level up to three years. The bone formation marker PINP Z-score was found to be significantly increased only after three and 24 months of TNF-α blocking therapy (*P *< 0.05). The bone resorption marker sCTX Z-score decreased significantly after three months (*P *< 0.001) and remained decreased during three years of treatment (Figure 1). The course of the absolute BTM values, analyzed separately for male and female patients because of gender differences in BTM, were in line with the results for the BTM Z-scores (data not shown).

**Figure 2:**
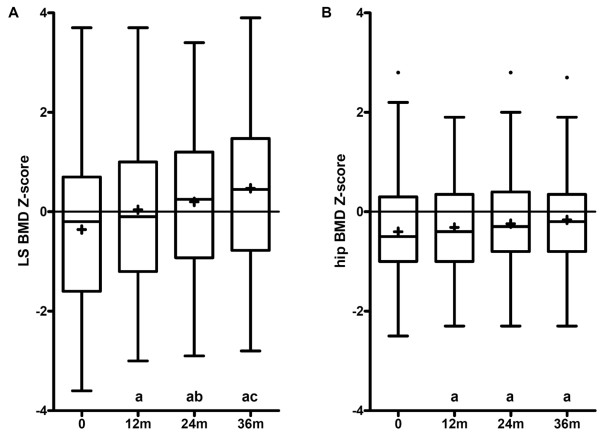
**The effect of three years of TNF-**α **blocking therapy on BMD in patients with AS**. **(A) **lumbar spine BMD. (**B) **hip BMD. Z-scores were calculated to correct for age and gender. Data were available for 92.4%, 93.1%, 89.6% and 64.6% of the 72 patients at 0, 12, 24 and 36 months, respectively. Box-and-whisker plots (Tukey): boxes indicate medians with interquartile ranges; + indicate means; whiskers indicate 1.5 times the interquartile distances; • indicate outliers. a: *P *< 0.001 compared to values recorded at baseline. b: *P *< 0.05 compared to values recorded at 12 months. c: *P *< 0.001 and *P *< 0.01 compared to values recorded at 12 and 24 months, respectively.

Lumbar spine and hip BMD Z-scores improved significantly after one year of TNF-α blocking therapy (*P *< 0.001). Subsequently, the lumbar spine BMD Z-score increased further after two and three years (*P *< 0.05 and *P *< 0.01, respectively). The hip BMD Z-score tended to increase further after two years (*P *= 0.050), but remained stable after two to three years of treatment (*P *= 0.780) (Figure 2).

### Predictive value of early change in bone turnover

Data of 105 AS patients were analyzed to investigate the predictive value of early change (0-3 months) in BTM for treatment discontinuation; 72 patients who continued versus 33 patients who discontinued their first TNF-α blocking agent because of inefficacy and/or adverse events. Patients who discontinued treatment due to other reasons (*n *= 6) were excluded from this analysis. Baseline to three months decrease in BASDAI, ASDAS_CRP_, patient's GDA, physician's GDA and sCTX Z-score were inversely associated with time to discontinuation of TNF-α blocking therapy in univariate Cox regression. Multivariate analysis showed that baseline to three months decrease in sCTX Z-score (HR: 0.394, 95% CI: 0.263 to 0.591), ASDAS_CRP _(HR: 0.488, 95% CI: 0.317 to 0.752), and physician's GDA (HR: 0.739, 95% CI: 0.600 to 0.909) were independent inversely related predictors of time to discontinuation of TNF-α blocking therapy (Table 3).

**Table 3:** Predictive value of early change in clinical and laboratory parameters for time to treatment discontinuation

	Univariate analysis		Multivariate analysis	
Assessment	HR (95% CI)	*P*-value	HR (95% CI)	*P*-value
Δ0 to 3 m BASDAI	0.672 (0.579 to 0.780)	0.000		*
Δ0 to 3 m ASDAS_CRP_	0.434 (0.327 to 0.577)	0.000	0.488 (0.317 to 0.752)	0.001
Δ0 to 3 m physician's GDA	0.643 (0.543 to 0.772)	0.000	0.739 (0.600 to 0.909)	0.004
Δ0 to 3 m patient's GDA	0.735 (0.641 to 0.844)	0.000		*
Δ0 to 3 m CRP	0.977 (0.954 to 1.002)	0.068		*
Δ0 to 3 m ESR	0.975 (0.950 to 1.001)	0.064		*
Δ0 to 3 m BALP Z-score	1.291 (0.969 to 1.720)	0.081		*
Δ0 to 3 m PINP Z-score	1.014 (0.688 to 1.493)	0.944		**
Δ0 to 3 m sCTX Z-score	0.513 (0.367 to 0.717)	0.000	0.394 (0.263 to 0.591)	0.000

Data of 105 AS patients were analyzed: 72 patients who continued versus 33 patients who discontinued their first TNF-α blocking agent because of inefficacy and/or adverse events.

Δ0 to 3 m, change from baseline to three months. See Table 1 for other abbreviations.

HR refers to the risk of anti-TNF-α treatment discontinuation per 1 grade or 1 point.

* The variable was not selected during multivariate Cox regression analysis (*P *≥ 0.05).

** The variable was not tested in multivariate Cox regression analysis because of a *P*-value > 0.3 in univariate analysis.

When the ASDAS was excluded from the analysis, baseline to three months decrease in sCTX Z-score (HR: 0.402, 95% CI: 0.273 to 0.593), BASDAI (HR: 0.702, 95% CI: 0.574 to 0.858), and physician's GDA (HR: 0.732, 95% CI: 0.602 to 0.891) were selected during multivariate analysis.

Since the number of female patients was relatively small, multivariate analysis using absolute BTM values was performed only in male patients. Baseline to three months decrease in sCTX (HR: 0.986, 95% CI: 0.979 to 0.993), BASDAI (HR: 0.707, 95% CI: 0.569 to 0.878) or alternatively, ASDAS_CRP _and physician's GDA (HR: 0.685, 95% CI: 0.522 to 0.898) were identified as independent inversely related predictors of time to treatment discontinuation.

The accuracy of baseline to three months change in sCTX Z-score to discriminate between patients who continued and discontinued TNF-α blocking therapy during the first three years was moderate, with an AUC of 0.741 (95% CI: 0.640 to 0.841), and comparable to the accuracy of early change in ASDAS_CRP _(AUC: 0.790, 95% CI: 0.699 to 0.880) or in physician's GDA (AUC: 0.730, 95% CI: 0.624 to 0.837).

In addition, baseline to three months change in sCTX Z-score was significantly associated with disease activity (BASDAI, ASDAS_CRP_, physician's and patient's GDA, ESR, and CRP), physical function (BASFI), spinal mobility (chest expansion), and quality of life (ASQoL) at last follow-up (defined as at three years of TNF-α blocking therapy or at the moment of treatment discontinuation) (Table 4).

**Table 4:** Correlations between early change in bone resorption marker sCTX and clinical assessments at last follow-up

	Δ0 to 3 sCTX Z-score	
**Assessment at last follow-up**^**a**^	correlation	*P*-value
BASDAI (range 0 to 10)	-0.388	0.000
ASDAS_CRP_	-0.463	0.000
Physician's GDA (range 0 to 10)	-0.321	0.001
Patient's GDA (range 0 to 10)	-0.260	0.010
ESR (mm/h)	-0.273	0.006
CRP (mg/l)	-0.288	0.004
BASFI (range 0 to 10)	-0.268	0.008
Chest expansion (cm)	0.260	0.010
Modified Schober test (cm)	-0.031	0.762
Occiput to wall distance (cm)	0.095	0.350
Lateral lumbar flexion L (cm)	0.096	0.346
Lateral lumbar flexion R (cm)	0.169	0.097
ASQoL (range 0 to 18)	-0.296	0.003

Data of 105 AS patients were analyzed.

See Tables 1 and 3 for abbreviations.

^a ^Defined as at three years of TNF-α blocking therapy or at the moment of treatment discontinuation

## Discussion

This is the first study that investigates the predictive value of early changes in bone turnover with regard to discontinuation of TNF-α blocking therapy in AS. Currently, in clinical practice, continuation of TNF-α blocking therapy is mainly based on subjective measures, such as the BASDAI and the global opinion of the patient and the physician. Recent studies showed the usefulness of the ASDAS as a more objective measure of disease activity [14-17,21]. However, a purely objective measure is still lacking in the evaluation process of TNF-α blocking therapy. The present analysis shows that a baseline to three months' decrease in sCTX Z-score was inversely related to time to discontinuation of TNF-α blocking therapy. Interestingly, sCTX Z-score remained a significant predictor of treatment discontinuation in the presence of ASDAS and physician's GDA, which underlines the value of sCTX in addition to the currently used measures. The accuracy of decrease in sCTX Z-score from baseline to three months in predicting treatment continuation was comparable to the moderate accuracy of early decrease in ASDAS or physician's GDA. Furthermore, early decrease in sCTX Z-score was significantly associated with good long-term response regarding disease activity, physical function, spinal mobility, and quality of life. Based on these results, early change in sCTX can be useful as an objective biomarker in the evaluation of TNF-α blocking therapy in patients with AS. A major advantage of BTM is that they can easily be measured in the blood of patients at different time points with relatively low costs. sCTX is widely available on automated immunoassay analysers or as ELISA. However, it is important to standardize the serum sample collection to reduce variability within and between patients [18].

Until now, several studies investigated the influence of TNF-α blocking therapy on bone formation and bone resorption up to one year of treatment [19-23]. The present study shows that three years of TNF-α blocking therapy resulted in a significant increase in the bone formation marker BALP, which plays a central role in the mineralization process of bone, at all time-points compared to baseline, while the effect on the bone formation marker PINP, a product of collagen formation, was found to be less evident. Furthermore, a significant decrease in the bone resorption marker sCTX, a product of collagen degradation, was found during three years of TNF-α blocking therapy. The significant increase in bone formation is in line with previous findings after one year of TNF-α blocking therapy [19-21]. Until now, no clear effect of TNF-α blocking therapy on bone resorption was reported [19,21-23]. In the present study, we had the unique availability of a healthy reference cohort on BTM, which allows us to correct the BTM levels of an individual AS patient for age and gender (using Z-scores). In this way, the rate of bone turnover can be studied without the confounding influence of age and gender, similar to the methodology of interpreting BMD. Nevertheless, our findings using the absolute BTM values (analysis split for gender) were in line with the results for the BTM Z-scores (data not shown).

The changes in BTM over time found in this study cannot be specifically attributed to TNF-α blocking therapy because a placebo group is lacking. Visvanathan *et al. *showed no significant changes in BALP or sCTX during 24 weeks of placebo treatment [23], which indicates that the present significant changes in BTM compared to baseline are the result of TNF-α blocking therapy. How these results fit into the pathogenesis of AS remains to be studied.

Importantly, the present results regarding BTM should not be extrapolated to any possible effect of TNF-α blocking therapy on radiographic progression in AS since no imaging method was used to measure new bone formation resulting in the formation of syndesmophytes and joint ankylosis. Furthermore, long-term observation is needed in order to see any effect of TNF-α blocking therapy on new bone formation in patients with AS.

Interestingly, both lumbar spine and hip BMD improved significantly during three years of TNF-α blocking therapy, which can be explained by the increase in mineralization and decrease in bone resorption. Alternatively, the increase in lumbar spine BMD may in part be confounded by the progression of formation of ligament calcifications and fusion of facet joints [5,29,30]. However, we expect that excessive bone formation will only have minor influence on the increase in lumbar spine BMD found in the present study, since previous studies reported a radiological progression of approximately one point in Modified Stoke Ankylosing Spondylitis Spinal Score (mSASSS; on a scale of 0 to 72) after two years of TNF-α blocking therapy [31-33]. Moreover, the improvement in lumbar spine and hip BMD after TNF-α blocking therapy is in line with previous findings [23,34].

## Conclusions

This prospective longitudinal observational cohort study shows that three years of TNF-α blocking therapy results in a bone turnover balance that favors bone formation (especially mineralization), in combination with continuous improvement of lumbar spine BMD. Furthermore, a baseline to three months' decrease in sCTX Z-score is identified as a significant inversely related predictor of time to treatment discontinuation, independent from ASDAS and physician's GDA. Based on these results, early change in the bone resorption marker sCTX seems useful as a purely objective biomarker in the evaluation of TNF-α blocking therapy in AS, in addition to the currently used more subjective measures.

## Abbreviations

AS: ankylosing spondylitis; ASAS: assessments in ankylosing spondylitis; ASDAS: ankylosing spondylitis disease activity score; ASQoL: ankylosing spondylitis quality of life; AUC: area under the curve; BALP: bone-specific alkaline phosphatase; BASDAI: Bath ankylosing spondylitis disease activity index; BASFI: Bath ankylosing spondylitis functional index; BMD: bone mineral density; BTM: bone turnover markers; CI: confidence interval; CRP: C-reactive protein; ECLIA: electrochemiluminescence immunoassay; ELISA: enzyme-linked immunosorbent assay; ESR: erythrocyte sedimentation rate; GDA: global disease activity; GEE: generalized estimating equations; GLAS: Groningen Leeuwarden Ankylosing Spondylitis; HR: hazard ratio; IBD: inflammatory bowel disease; IE-CV: inter-assay coefficient of variation; MCL: Medical Center Leeuwarden; MRI: magnetic resonance imaging; OC: osteocalcin; PINP: procollagen type 1 N-terminal peptide; RCT: randomized controlled trial; RIA: radioimmunoassay; ROC: receiver operating characteristic; sCTX: serum type I collagen C-telopeptide; SD: standard deviation; sNTX: serum type I collagen N-telopeptide; TNF-α: tumor necrosis factor alpha; UMCG: University Medical Center Groningen; WHO: World Health Organization

## Competing interests

AS and PH have received unrestricted research grants from Wyeth. EB has received unrestricted research grants from Abbott, Schering-Plough, and Wyeth. The other authors declare that they have no competing interests.

## Authors' contributions

SA participated in the design of the study, performed the statistical analysis and interpretation of data, and drafted the manuscript. AS and EB participated in the design of the study, performed the acquisition of data, and critically revised the manuscript. PH, ML and RB contributed to the acquisition of clinical data and critically revised the manuscript. CK participated in the design of the study and critically revised the manuscript. HG contributed to the statistical analysis and interpretation of data and critically revised the manuscript. EV participated in the design of the study, contributed to the statistical analysis and interpretation of data and critically revised the manuscript. All authors approved the final manuscript.
